# Antibiotic prophylaxis for ophthalmia neonatorum in Italy: results from a national survey and the Italian intersociety new position statements

**DOI:** 10.1186/s13052-023-01507-7

**Published:** 2023-09-11

**Authors:** Vito Mondì, Chryssoula Tzialla, Salvatore Aversa, Daniele Merazzi, Stefano Martinelli, Gabriella Araimo, Luca Massenzi, Giacomo Cavallaro, Luigi Gagliardi, Fiammetta Piersigilli, Mario Giuffrè, Simona Lozzi, Paolo Manzoni, Fabio Mosca, Irene Cetin, Vito Trojano, Herbert Valensise, Nicola Colacurci, Luigi Orfeo, Cinzia Auriti

**Affiliations:** 1 Neonatology and Neonatal Intensive Care Unit, Policlinico Casilino, Via Casilina 1049, Rome, Italy; 2 Neonatal and Pediatric Unit, ASST Pavia, Via Volturno 14, Voghera, Italy; 3 Neonatal Intensive Care Unit, Children’s Hospital, ASST Spedali Civili, Piazzale Spedali Civili 1, Brescia, Italy; 4 Division of Neonatology, ‘Valduce’ Hospital, Via Dante Alighieri 11, Como, Italy; 5 Neonatal Intensive Care Unit, ASST Grande Ospedale Metropolitano Niguarda, Piazza Dell’Ospedale Maggiore 3, Milan, Italy; 6 Neonatal Intensive Care Unit, Fondazione IRCCS Ca’ Granda Ospedale Maggiore Policlinico, Via Francesco Sforza 28, Milan, Italy; 7 Division of Neonatology, Central Teaching Hospital of Bolzano, Via Lorenz Böhler 5, Bolzano, Italy; 8 Division of Neonatology and Pediatrics, Versilia Hospital, Azienda USL Toscana Nord Ovest, SS1 335 ViareggioPisa, Italy; 9 Section of Neonatology, Cliniques Universitaires Saint Luc, Université Catholique de Louvain, Avenue Hippocrate 10, Brussels, Belgium; 10 Neonatal Intensive Care Unit, A.U.O.P. ‘P. Giaccone,’ Department of Health Promotion, Mother and Child Care, Internal Medicine and Medical Specialties ‘G. D’Alessandro’, University of Palermo, Via del Vespro 129, Palermo, Italy; 11 Neonatal Intensive Care Unit, Medical and Surgical Department of Fetus, Newborn and Infant – “Bambino Gesù” Children’s Hospital IRCCS,, Piazza Di Sant’Onofrio 4, 00165 Rome, Italy; 12 Department of Maternal, Neonatal and Infant Medicine, University Hospital “Degli Infermi”, Via Dei Ponderanesi 2, Ponderano, Italy; 13 Department of Clinical Sciences and Community Health, University of Milan, Via Della Commenda 19, Milan, Italy; 14 Department of BioMedical and Clinical Sciences, University of Milan, Via Givan Battista Grassi 74, Milan, Italy; 15 Department of Obstetrics and Gynecology, Hospital V. Buzzi, ASST Fatebenefratelli Sacco, Via Lodovico Castelvetro 32, Milan, Italy; 16 Department of Obstetrics and Gynaecology, Mater Dei Hospital, Via Samuel F. Hahnemann 10, Bari, Italy; 17 Department of Surgical Sciences, University of Rome Tor Vergata, Via Montpellier, 1, Rome, Italy; 18 Obstetrics and Gynecology, Policlinico Casilino, Via Casilina 1049, Rome, Italy; 19 Department of Woman, Child and General and Specialized Surgery, Obstetrics and Gynecology Unit, University of Campania “Luigi Vanvitelli”, Via Luigi De Crecchio 2, Naples, Italy; 20 Neonatal Intensive Care Unit, Fatebenefratelli Isola Tiberina – Gemelli Isola, Via Di Ponte Quattro Capi 39, Rome, Italy; 21 Villa Margherita Private Clinic, Via Di Villa Massimo 48, 00161 Rome, Italy

**Keywords:** Ophthalmia Neonatorum, *Neisseria gonorrhoeae*, *Chlamydia trachomatis*, Prophylaxis

## Abstract

**Background:**

Ophthalmia neonatorum is an acute conjunctivitis that occurs in newborns within the first month of life. The most serious infections are due to *Chlamydia trachomatis* and *Neisseria gonorrhoeae*, that may cause permanent damages. The use of ophthalmic prophylaxis varies widely around the world, according to the different health and socio-economic contexts. To date in Italy there is no a clear legislation regarding ophthalmia neonatorum prophylaxis at birth.

**Methods:**

We invited all birth centers in Italy to carry out a retrospective survey relating the last three years. We collected data regarding demographics of neonates, drugs used for ophthalmic prophylaxis and results of the screening of pregnant women for *Chlamydia trachomatis* and *Neisseria gonorrhoeae* vaginal infections.

**Results:**

Among 419 birth centers, 302 (72,1%) responded to the survey. Overall 1041384 neonates, 82,3% of those born in the three years considered, received ophthalmic prophylaxis. Only 4,585 (0,4%) of them received one of the drugs recommended by the WHO. The Centers that participated to the survey reported 12 episodes of Chlamydial conjunctivitis and no Gonococcal infection in the three years. Only 38% of the Centers performed vaginal swabs to pregnant women: 2,6% screened only for *Neisseria*, 9,6% only for *Chlamydia* and 25,8% for both germs.

**Conclusions:**

The data obtained from the survey showed a low incidence of neonatal conjunctivitis due to either *Neisseria gonorrhoeae* or *Chlamydia trachomatis* in Italy. Due to the lack of legislation regulating the prophylaxis of ophthalmia neonatorum in newborns, the Italian Society of Neonatology, the Italian Society of Obstetrics and Gynecology and the Italian Society of Perinatal Medicine have recently issued new recommendations on this topic.

## Background

Ophthalmia neonatorum (ON) is an acute conjunctivitis that occurs in newborns within the first month of life [1–3]. Originally this term only referred to cases caused by *Neisseria gonorrhoeae*, but currently it includes any conjunctivitis in this age group. *Neisseria gonorrhoeae* and *Chlamydia trachomatis* cause the most serious infections, with possible severe complications such as corneal ulceration, corneal perforation or permanent blindness. *Neisseria gonorrhoeae* conjunctivitis now accounts for less than 1% of reported cases of ON in the United States, while *Chlamydia trachomatis* infections ranges from 2 to 40%. Other bacteria such as *Staphylococcus*, *Streptococcu*s, *Hemophilus* and some Gram-negative bacteria account for 30% to 50% of cases [2]. The epidemiology of ON due to *Neisseria gonorrhoeae* dramatically decreased in Germany, from 10 to 0,3% [4], after Carl Siegmund Franz Credè (1819–1892) introduced, in the late nineteenth century, the prophylaxis with 2% silver nitrate solution instilled in the conjunctiva of newborns, to prevent the gonococcal conjunctivitis [5]. Before this procedure *Neisseria gonorrhoeae* was the primary cause of neonatal blindness (60–73%) in Germany [6]. Subsequently, the practice of topical prophylaxis with 2% silver nitrate spread rapidly around the world.

The worldwide incidence of gonococcal conjunctivitis currently varies from 2 to 23%. This variability depends on the specific socio-economic context and on the level of maternal and prenatal care [7]. In the United States, from 2013 to 2017, the estimated incidence of gonococcal conjunctivitis was 0,4 cases/100 000 live births per year [8, 9] while that of chlamydial conjunctivitis was 2,1/100 000 live births per year [9]. Unfortunately, the cases of neonatal conjunctivitis due to *Neisseria gonorrhoeae* or *Chlamydia trachomatis* in Italy are not reported by the national surveillance system of the Italian Institute of Health; therefore, there are no available data on the incidence of these infections in Italian newborns.

Both *Neisseria gonorrhoeae* and *Chlamydia trachomatis* infections are contracted during childbirth by the contact of newborn with the infected endocervical secretions. The neonate born to an infected mother has a 30% to 50% chance to develop conjunctivitis [10]. Untreated or inappropriately treated gonococcal conjunctivitis may result in corneal perforation and vision loss in less than 24 hours [11]. Untreated Chlamydial conjunctivitis can be associated with corneal and conjunctival scarring, hemorrhagic conjunctivitis, and rarely, loss of vision [12–14].

Due to the potential complications related to gonococcal and chlamydial conjunctivitis in newborns, the World Health Organization (WHO) guidelines [15] recommend topical ophthalmic prophylaxis for all newborns, immediately after birth, with one of the following treatments:

- Tetracycline hydrochloride 1% eye ointment;
- Erythromycin 0,5% eye ointment;
- Povidone iodine 2,5% solution (water-based);
- Silver nitrate 1% solution;
- Chloramphenicol 1% eye ointment.

To date in Italy the area of ON and its prophylaxis shows several gaps of knowledge and inconsistencies in practice and management.

First, prophylaxis is not regulated by any national legislation, since the old law of 1940, which established its obligation, has been repealed and never updated.

Second, no nationwide, recent data regarding frequency of use and type of ophthalmic prophylaxis at birth in Italy are available.

Third, we lack epidemiological data on the actual incidence of ON in neonates in Italy.

In order to address these pending questions, we conducted a nationwide questionnaire survey, to collect data regarding the prophylaxis of ON, the type of antibiotics used and the frequency of the screening for *Chlamydia trachomatis* and *Neisseria gonorrhoeae* vaginal infections in pregnant women in Italy, as well as the actual incidence of ON in the nurseries.

## Methods

### Design and settings

In 2021 a questionnaire in electronic format was sent to all birth centers in Italy with the questions showed in the Table 1. The questionnaire sent out requests data on all infants born in each center between 1^st^ of January 2018 and 31^st^ of December 2020. Responding Centers were divided according to the Italian Ministry of Health’s instructions regarding the number of assisted births per year [16] in four different groups: centers assisting less than 500 births/year, between 500 to 999 births/year, between 1000 to 2499 births/year and more than 2500 births/year.

**Table 1:** Survey questions

• Which was the total number of newborn in your center from the 1^st^ January 2018 to the 31 of December 2020 ?
• Did you administer routinely conjunctival antibiotics as prophylaxis to newborns at birth?
• Which drugs do you use routinely?
• How is the preparation of the drug: single or multi doses?
• How many cases of ON do you have observed during the three-year period and which pathogen was responsible?
• Do you collect maternal data relating to chlamydial and gonococcal infections during pregnancy?

Questionnaire sent to birth centers in electronic format

### Participants and study procedures

The following data were collected on each birth centers: number of births between 1^st^ of January 2018 and 31^st^ of December 2020, performance of eye prophylaxis, drug administered, type of packaging, number of ON cases in the same three-year period, maternal data on screening for *Chlamydia trachomatis* and/or *Neisseria gonorrhoeae* performed during pregnancy. Chlamydial or gonococcal ON were defined to assure consistency among sites. ON is defined as conjunctivitis occurring within the first 4 weeks after birth. Gonococcal ON has an incubation period of 2–5 days, with ocular swab positive for *Neisseria gonorrhoeae*. Chlamydial ON has an incubation period of 5–12 days with ocular swab positive for *Chlamydia trachomatis* [17]. In addition, information was obtained on the geographic location of the birth centers.

### Data sources

Information on how respondents are recruited to SurveyMonkey is available here: http://www.surveymonkey.com/mp/audience. A neonatologist from each department anonymously entered the answers to the questions. All data were anonymized. The answers to the questions were only available to persons appointed as data processors. Those who answered the questions could only view the answers from their own hospital. Although we were not able to perform external data validation at each site, procedures were implemented to minimize data entry errors.

### Statistical analysis

Data are presented as descriptive analysis, means and proportions, and have been processed by Microsoft Excel version 16.68 for Mac.

### Ethics statement

In Italy, approval by the ethics committee is not required in surveys that do not require specific data of each patient, completed by medical staff.

### Funding source

This study did not receive any funding.

## Results

### Demographics

**Fig. 1:**
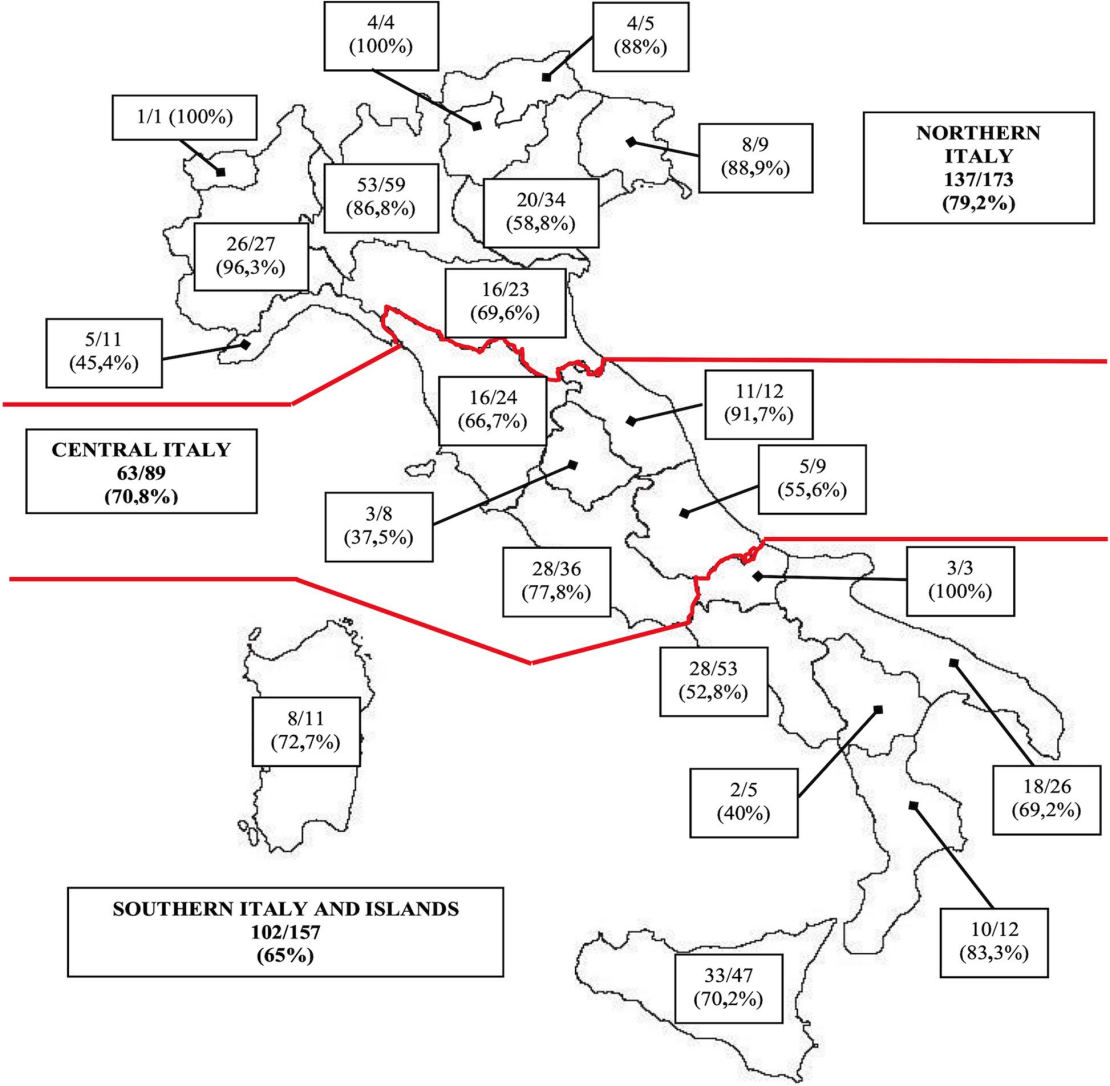
Geographic distribution of responding centers. Regional distribution of the Italian centers that have replied to the questionnaire

In 2021, all Italian birth Centers were approached and sent the questionnaire. Some 72% of them took the survey [302/419: 137/ 173 (79,2%) in Northern, 63/89 (70,8%) in Central and 102/157 (65%) in Southern Italy]. Among the centers assisting less than 500 births/year, 50/103 (48,5%) answered to the survey, among those assisting between 500 to 999 births/year 123/170 (72,3%) centers answered, among those assisting between 1000 to 2499 births/year 112/127 (88,2%) centers answered and those assisting more than 2500 births year 17/19 (89,5%). We did not observe any difference in terms of adherence to the survey between centers of North, Centre, and South of Italy nor between regions. Figure 1 and Table 2 show the distribution of the results clustered by Italian Regions.

**Table 2:** Responding birth centers

Birth per year	Neonatal wards available	Neonatal wards answered	%
**Italy**			
< 500	103	50	48,5%
500–999	170	123	72,3%
1000–2499	127	112	88,2%
> 2500	19	17	89,5%
**Northern Italy**			
**Valle D’Aosta**			
500–999	1	1	100%
**Piedmont**			
< 500	7	6	85,7%
500–999	13	13	100%
1000–2499	6	6	100%
> 2500	1	1	100%
**Liguria**			
< 500	4	0	0%
500–999	4	4	100%
1000–2499	3	1	33,3%
**Lombardy**			
< 500	9	6	66,7%
500–999	23	22	95,7%
1000–2499	21	21	100%
> 2500	6	4	66,7%
**Autonomous Province of Bolzano/South tyrol**			
< 500	1	0	0%
500–999	2	2	100%
1000–2499	2	2	100%
**Autonomous Province of Trento**			
< 500	2	2	100%
1000–2499	2	2	100%
**Veneto**			
< 500	7	2	28,6%
500–999	14	7	50%
1000–2499	12	10	83,3%
> 2500	1	1	100%
**Friuli Venezia-Giulia**			
< 500	1	1	100%
500–999	5	4	80%
1000–2499	3	3	100%
**Emilia-Romagna**			
< 500	7	2	28,6%
500–999	5	3	60%
1000–2499	6	6	100%
> 2500	5	5	100%
**Central Italy**			
**Tuscany**			
< 500	4	1	25%
500–999	11	8	72,8%
1000–2499	8	6	75%
> 2500	1	1	100%
**Latium**			
< 500	12	7	58,3%
500–999	12	9	75%
1000–2499	8	8	100%
> 2500	4	4	100%
**Umbria**			
< 500	3	1	33,3%
500–999	3	1	33,3%
1000–2499	2	1	50%
**Marche**			
< 500	3	2	66,7%
500–999	6	6	100%
1000–2499	3	3	100%
**Abruzzo**			
< 500	2	2	100%
500–999	5	2	40%
1000–2499	2	1	50%
**Southern Italy and Islands**			
**Campania**			
< 500	13	4	30,8%
500–999	23	11	47,9%
1000–2499	16	12	75%
> 2500	1	1	100%
**Molise**			
< 500	2	2	100%
500–999	1	1	100%
**Apulia**			
< 500	4	2	50%
500–999	13	8	61,3%
1000–2499	9	8	88,9%
**Basilicata**			
< 500	2	0	0%
500–999	2	2	100%
1000–2499	1	0	0%
**Calabria**			
< 500	2	1	50%
500–999	5	4	80%
1000–2499	5	5	100%
**Sicily**			
< 500	14	7	50%
500–999	18	12	66,7%
1000–2499	15	14	93,3%
**Sardinia**			
< 500	4	2	50%
500–999	4	3	75%
1000–2499	3	3	100%

Number of neonatal wards of the Italian regions answered the survey, divided according to the number of births in 2020 (20), divided in 4 differents group (< 500/year; 500–999/year; 1000–2499/year;  > 2500/year)

### Ophthalmic prophylaxis

During the study period all neonates born in the centers participating to the survey (which correspond to 82,3% of total births in Italy in the same period [18–20]) underwent ophthalmic antibiotic prophylaxis. Newborns who received one of the drugs recommended by the WHO (povidone iodine 2,5% solution) were 4585/1041384 (0,4%). Chloramphenicol and tetracycline were administered to 37095/1041384 (3,6%) and to 46193/1041384 (4,4%) of newborns, respectively, but as topical antibiotic solution, combined with other drugs and not as eye ointment, as recommended by the WHO. Topical tobramycin was administered in 474418/1041384 (45,6%) newborns and gentamycin in 202937/1041384 (19,5%).

**Fig. 2:**
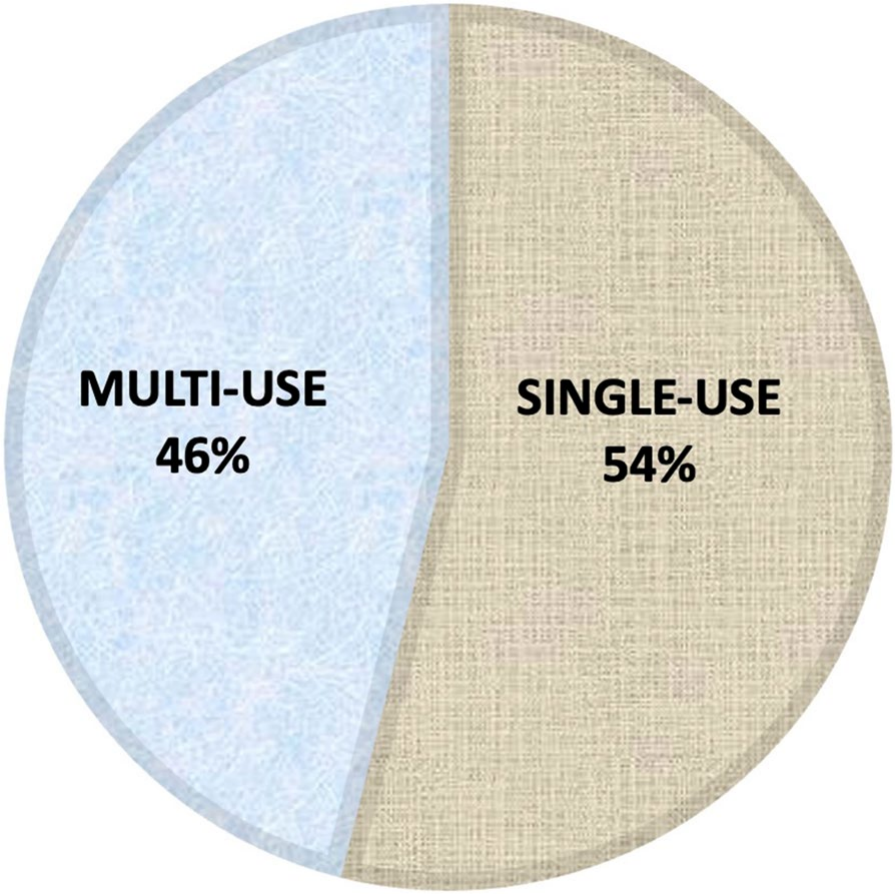
Drug packaging. Percentage of infants who have received eye drops from single-use or multi-purpose pharmaceutical packaging for ophthalmic prophylaxis

**Fig. 3:**
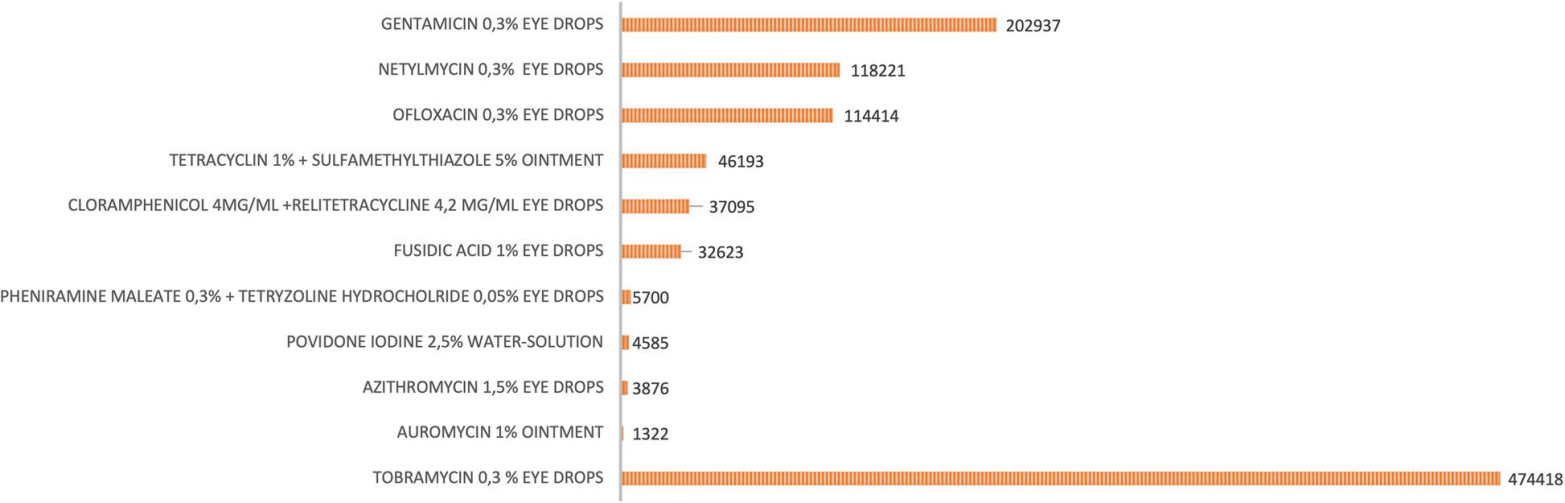
Drugs used for the ophthalmic prophylaxis. Number of neonates who received the different antibiotic molecules in infants observed with the survey

The WHO recommendation invites to use a single-use antibiotic ointment. The medications used for prophylaxis of the Italian newborns included in the survey were in double antibiotic solution, in multi-use packaging and not prepared as single-use antibiotic ointment (Fig. 2). Therefore, 1036799/1041384 newborns (99,6%) received a suboptimal ophthalmic prophylaxis. Figure 3 shows the drugs used for the prophylaxis in our cohort of newborns.

### Ophthalmia neonatorum

Overall, 12 cases of *Chlamydia trachomatis* conjunctivitis were reported (incidence rate 0,001%), all occurring in only six neonatal units (2%) out of 302 participating to the survey reported *Neisseria gonorrhoeae* infection was never reported.

### Pregnancy data

**Fig. 4:**
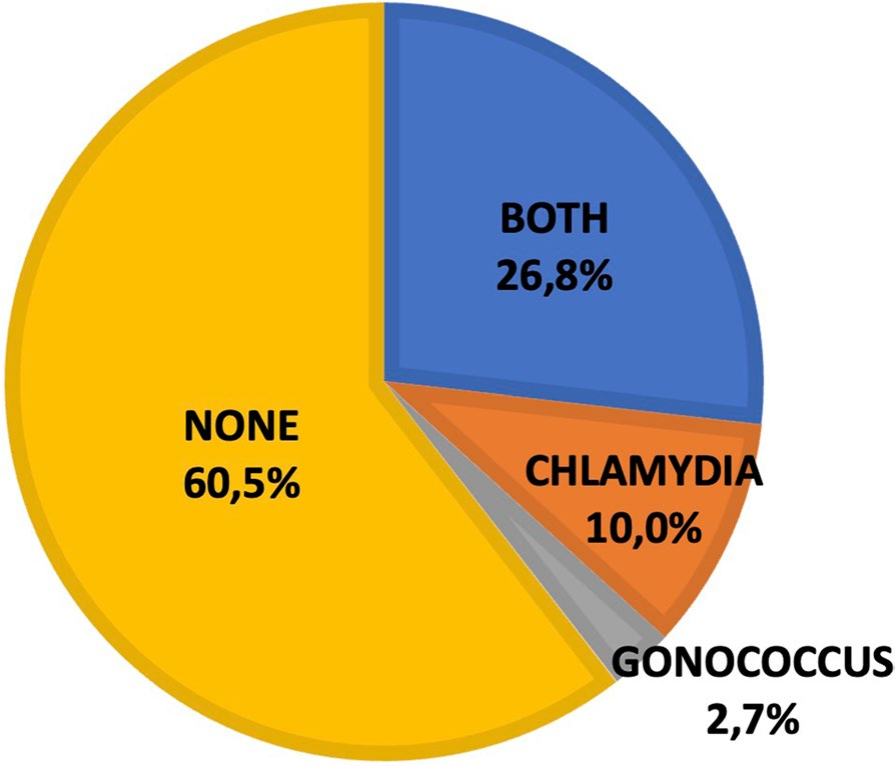
Information on maternal screening. Frequency of maternal vaginal screening for Chlamydia trachomatis and Neisseria gonorrhoeae in Italian centers participating in the survey

One hundred and fifteen centers (39,5%) performed screening of sexually transmitted diseases via vaginal swabs: 8 (2,7%) centers for gonococcal infections, 29 (10%) for chlamydial infections and 78 (26,8%) for both germs (Fig. 4).

## Discussion

In Italy there is no clear legislation regarding ophthalmia neonatorum prophylaxis at birth. The law regulating the administration of antibiotic prophylaxis to newborns at birth dates back to 1940 [21] and has been repealed in 1975 [22]. Despite this gap in legislation on this subject, the administration of conjunctival antibiotics to newborns in Italy is still routinely carried out in all birth centers.

In Italy, the National Surveillance System of the National Health Institute does not collect data on the incidence of chlamydial or gonococcal conjunctivitis in the neonatal period. The data from our national survey shed a light on the incidence of neonatal conjunctivitis and on the current prophylactic practices in Italy, at the same time underpinning an urgent need to update the Italian scientific societies’ recommendations regarding the administration of ophthalmic prophylaxis.

It is somewhat concerning that in Italy 99,6% of all newborns did not receive at birth the ophthalmic prophylaxis according to WHO recommendations, despite an extremely wide use of ophthalmic prophylaxis. Although this clear inconsistency, no cases of gonococcal and very few cases of chlamydial conjunctivitis in newborns have been reported period of the study. These low incidences of conjunctivitis recorded by our survey may be a limitation of our report. However, it must be considered that a high number of birth points participated in our survey, in most of which infants are followed up for routine checkups even after discharge. This allows the identification of conjunctivitis at least in the first 10 days of life. In addition, the severe conjunctivitis may require hospitalization, and in Italy all infants must be admitted to Neonatal Units. Surely, more complete data would have been obtained by involving all primary care pediatricians and all pediatric ophthalmology units; in contrast, we would have had a lower survey participation rate.

Several European countries no longer administer universal ocular prophylaxis but recommend the screening and treatment of sexually transmitted diseases in pregnant women at high risk. The Canadian Pediatric Society does not recommend universal prophylaxis with erythromycin but promote epidemiological investigations to estimate the incidence of ON [23]. However, in countries where ophthalmic antibiotic prophylaxis is still performed, there is no agreement on which drugs to use. In the United States, only the 0,5% erythromycin ophthalmic ointment is available among the drugs recommended by the WHO. Other medications, such as tetracycline ophthalmic ointment and silver nitrate, are no longer available and gentamicin was reported as responsible for chemical conjunctivitis [24]. In Brazil, the use of povidone iodine 2,5%, erythromycin 0,5%, tetracycline 1%, or silver nitrate 1% is recommended as rescue [25], in Chile chloramphenicol 0,5% or erythromycin 0,5% are used [26], and in Spain ophthalmic prophylaxis with erythromycin 0,5% or tetracycline 1% ointment is performed only in newborns delivered by vaginal route [27]. Whichever the antibiotic chosen, it is mandatory to use preparations with a single active ingredient. Furthermore, it is necessary to administer the drug exclusively using a single-dose package, to prevent any spread of conjunctival infections and to allow proper storage of the drug.

To prevent ON, the Center for Disease Control and Prevention (CDC), recommends that all pregnant women with risk factors for sexually transmitted diseases and their partners should be screened for *Neisseria gonorrhoeae* at the first prenatal visit [28]. Pregnant women who remain at high risk for gonococcal and chlamydial infection should be tested again during the third trimester to prevent maternal postnatal complications and a gonococcal infection in the neonate [29].

## Conclusion

In the light of the above mentioned evidence, and taking into account the scenario that our data have disclosed, the low incidence in Italy of neonatal conjunctivitis from *Chlamydia trachomatis* and *Neisseria gonorrhoeae*, and the current national shortage of drugs recommended by the CDC and the WHO, the Italian Society of Neonatology (SIN), the Italian Society of Gynecology and Obstetrics (SIGO) and the Italian Society of Perinatal Medicine (SIMP), have deemed necessary to issue a position statement [30], with the aim of providing shared indications for the best and appropriate preventive approach of neonatal conjunctivitis contracted during delivery, thus overcoming the absence of a specific regulation on the mandatory nature of ocular prophylaxis in the newborn.

The two objectives of the position statement are the following:

1. To standardize the prophylactic procedures throughout Italy, at the same time assessing the actual need to perform ophthalmic prophylaxis to all birth cohorts based on the data coming from Italian birth centers
2. To avoid administering useless antibiotics to infants, which may be in turn harmful when used without a precise indication.

For this initiative to be successful, it is essential to have a multidisciplinary approach, with the obstetrical, neonatological and nursing staff working together to have primary prevention activities to be started from the earliest stages of pregnancy.

The Intersociety document does not recommend anymore the administration of the ophthalmic antibiotic prophylaxis at birth to all newborns, but only to neonates born from unattended pregnancies (defined as less than three visits performed during pregnancy) or unscreened pregnant women at risk for sexually transmitted diseases. Neonates requiring ophthalmic prophylaxis should receive immediately after birth, either 0,5% erythromycin or 1% tetracycline or 1% chloramphenicol ophthalmic ointment.

Moreover, a vulvovaginal swab for *Neisseria gonorrhoeae* and *Chlamydia trachomatis* is recommended for all pregnant women at risk for sexually transmitted diseases at the first prenatal care visit. The document recommends against administering ophthalmic antibiotic prophylaxis at birth to neonates born from a mother with a vaginal swab positive for *Chlamydia trachomatis* regardless of treatment during pregnancy or with a vaginal swab positive for *Neisseria gonorrhoeae*, but adequately treated during pregnancy.

Finally, symptomatic neonates born to a mother positive for *Chlamydia trachomatis,* regardless of treatment during pregnancy, and asymptomatic/symptomatic neonates born to a mother positive for *Neisseria gonorrhoeae* not treated or inadequately treated during pregnancy should be treated with systemic antibiotic therapy.

## Data Availability

The datasets used and analyzed during the current study are available from the corresponding author on reasonable request.

## Abbreviations

ON: Ophthalmia neonatorum
WHO: World health organization
CDC: Center for disease control and prevention
SIN: Italian society of neonatology
SIGO: Italian society of gynecology and obstetrics
SIMP: Italian society of perinatal medicine
